# Extract from a Letter from Dr. C. Dumreicher

**Published:** 1862-05

**Authors:** C. Dumreicher

**Affiliations:** Cumberland, Md.


### *Extract from a Letter from Dr. Dumreicher.*

CUMBERLAND, MD., *April* 18, 1862.

DEAR DOCTOR:—About six weeks ago, I was sent by Gen. Rosencrans to this place, there being a great want of surgeons, and the number of sick soldiers from the late Gen. Lander's division increasing very rapidly. I arrived here and found a great deal of work to do. 1500 patients and very limited accommodations. I worked hard for two weeks; had many cases of typhoid fever, pneumonia, typhoid pneumonia, rheumatism, &c. I succeeded better than I could expect; and was much pleased by receiving a very flattering official notice from the Med. Inspector and Med. Director of the Department. There is quite an epidemic around here of a peculiar disease of the throat,—something between diphtheria and the sore throat frequently accompanying scarlatina. It is hard to manage; and characterized by *extreme* prostration. I had such an attack myself, and was very ill. I am told that this disease has been quite fatal here. Respectfully and truly yours,

C. DUMREICHER.

